# The link between attitudes toward probationers and job burnout in Turkish probation officers

**DOI:** 10.1002/jcop.22673

**Published:** 2021-07-16

**Authors:** Ayşe E. Ersayan, Banu Çankaya, Gizem Erdem, Nick J. Broers, Corine de Ruiter

**Affiliations:** ^1^ Department of Clinical and Psychological Science Maastricht University Maastricht The Netherlands; ^2^ Department of Psychology Koç University Istanbul Turkey; ^3^ Department of Psychology MEF University Istanbul Turkey; ^4^ Department of Methodology and Statistics Maastricht University Maastricht The Netherlands

**Keywords:** attitudes toward probationers, individual characteristics, job burnout, probation officers

## Abstract

The goal of the current study was to investigate individual‐level factors associated with job burnout among probation officers (POs) and, specifically, to examine if attitudes toward probationers were linked with job burnout in the context of the recently established probation system in Turkey. Participants (*N* = 115) were recruited from a probation office in Istanbul. Job burnout was assessed via three components: emotional exhaustion, depersonalization, and professional accomplishment. Results of structural equation modeling indicated that more favorable attitudes toward probationers were related to a lower sense of depersonalization and higher experience of professional accomplishment. However, POs’ attitudes toward probationers were not associated with emotional exhaustion. Our findings are discussed in light of the present empirical literature on the contextual factors influential in job burnout. Practical implications for burnout prevention point to the potential effectiveness of working on attitudes among POs toward the people they supervise.

## 1 INTRODUCTION

Probation officers (POs) play a critical role in the probation system. They are expected to fulfill competing public safety, punishment, and rehabilitation goals while managing large caseloads, often with limited training opportunities for a professional qualification (Pitts, 2007). Given the demands of their work and limited resources in meeting the workload, POs are vulnerable to job burnout (White et al., 2015; Whitehead, 1985). Over the past four decades, several studies have investigated the job‐related factors that are associated with job burnout in POs, including role conflict (Allard et al., 2003), job stress (Griffin et al., 2010; Lewis et al., 2013), and organizational climate (Gayman & Bradley, 2013).

Fewer studies have focused on the individual characteristics of the POs concerning job burnout compared to studies on job‐related characteristics. The impact of socio‐demographic factors, such as age (Gayman et al., 2018), work experience (Andersen et al., 2017), and education level (Keinan & Malach‐Pines, 2007), has been reported in several studies, but results have been mixed. Among the few studies focusing on the link between individual characteristics and job burnout among POs, attitudes have attracted the least attention albeit being a modifiable target of intervention. Only two studies (Gallavan & Newman, 2013; Schaefer & Williams, 2018)—to our knowledge—have been conducted to understand whether attitudes toward adult probationers are linked to PO burnout.

One of the two studies was conducted with 101 correctional mental health professionals (Gallavan & Newman, 2013). Favorable attitudes toward prisoners were found to be related to positive work experience, which was defined by professional accomplishment and feelings of pleasure associated with work (Gallavan & Newman, 2013). On the other hand, dis/favorable attitudes toward prisoners were not related to negative work experience defined by depersonalization, emotional exhaustion, and secondary traumatization (Gallavan & Newman, 2013). Another study with 75 parole and POs in a metropolitan city in Australia examined associations of attitudes toward probationers with burnout (Schaefer & Williams, 2018). Results indicated associations of negative attitudes with increased exhaustion, increased depersonalization, and decreased personal accomplishment.

The aforementioned studies examined job burnout in a Western context where the probation systems are well established, decentralized, provide supervision and other resources to the officers. In the current study, we aim to examine both socio‐demographic characteristics and attitudes toward probationers as potential correlates of PO job burnout in the Turkish criminal justice system. The Turkish probation system was established in 2005 (Yavuz, 2012) in response to the accelerating incarceration rates across the country. The novelty of the probation system, the ongoing ambiguity of role definitions as well as procedures (Aslanyürek Zorlu, 2014), the centrality of the system with direct control by the Ministry of Justice and the Turkish state (Kavur, 2020), and the lack of resources for rehabilitation services (Erdem et al., 2019; Tuncer et al., 2020) make the Turkish case a unique context to explore burnout and attitudes toward probationers.

### 1.1 Job burnout

Job burnout is a state of physical and psychological stress in response to the emotional and professional demands of a job. It has been conceptualized as comprising three interrelated core components (Maslach et al., 2001). *Emotional exhaustion* is the stress component of burnout; it is identified by a lack of enthusiasm for work and emotional depletion. *Depersonalization* refers to distancing oneself from work and the clients one serves. *Reduced personal accomplishment* is characterized by feelings of inefficacy in one's personal and professional life.

Maslach et al. (2001) reviewed numerous studies on correlates of job burnout across different helping professions and suggested that correlates can be categorized into *situational* and *individual* factors. In an attempt to understand situational factors, the authors defined three subfactors: *job characteristics, occupational characteristics*, and *organizational characteristics*. A wealth of research, conducted over 40 years, has provided consistent results on situational factors as correlates of job burnout. Job characteristics, such as role ambiguity, role conflict, absence of social support, lack of feedback, limited control, and autonomy were correlated with emotional exhaustion (Pitts, 2007; White et al., 2015). In a study of 825 probation and parole officers, Gayman and Bradley (2013) found role ambiguity, role conflict, role overload, and work stress to be significant predictors of job burnout. Among occupational characteristics, for example, emotional challenges at work accounted for 12% of the variance in emotional exhaustion in a sample of 1242 adults with various service occupations, such as nurses, waiters, call center employees, and social workers (Zapf et al., 2001). Gayman et al. (2018) found that having more supervisees with mental health problems predicts more emotional exhaustion among probation and parole officers (*N* = 798). Organizational characteristics, such as not being allowed to provide input for decision making were also significantly correlated with increased burnout among 272 prison staff (Lambert et al., 2010).

In their model, Maslach et al. (2001) proposed that individual factors also play a role in burnout and these are composed of *demographics, personality characteristics*, and *job attitudes*. There has been limited research on what individual‐level factors are associated with burnout among POs (Gallavan & Newman, 2013). In an attempt to address this gap, the current study focuses on an individual‐level factor that may be linked to POs' job burnout, that is, their attitudes toward probationers (Gallavan & Newman, 2013). We investigate attitudes toward adult probationers in relation to PO job burnout. We incorporate demographic factors in our study as control variables given mixed findings regarding their associations with burnout in previous studies across various countries (e.g., EU countries, Canada, Australia, and the United States; Purvanova & Muros, 2010).

### 1.2 Attitudes toward service recipients and job burnout

An attitude is a subjective evaluation that encompasses emotions, thoughts, beliefs, and behaviors toward something, someone, some issue, or some event (Breckler, 1984; Petty & Wegener, 1997). Human service professionals' attitudes toward service recipients have been investigated concerning job burnout, but these studies are few in number in a criminal justice system context. In studies among other human service professionals, negative attitudes toward patient care were examined in association with burnout levels among pediatric residents (Baer et al., 2017) and medical students (Dyrbye et al., 2010). Findings indicated positive associations between negative attitudes toward patients and burnout levels (Baer et al., 2017; Dyrbye et al., 2010). Another study on community service providers’ attitudes toward intellectual disability and mental illness (Tartakovsky et al., 2013) revealed that empowerment‐oriented attitudes, reported a sense of similarity, and negative attitudes toward social exclusion, predicted low burnout in‐service staff, including managers, social workers, and support workers. In tutors of children with autism, high levels of negative attitudes toward autism, assessed with both implicit and explicit measures, were associated with higher burnout (Kelly & Barnes‐Holmes, 2013). Holmqvist and Jeanneau (2006) found that more negative attitudes toward psychiatric patients were related to increased burnout among mental health professionals.

Studies have documented that negative attitudes toward prisoners are common in the correctional system, mainly among correctional officers, attorneys, and prison rehabilitation teams (Melvin et al., 1985; Ortet‐Fabregat et al., 1993). The predictors of negative attitudes (i.e., years of work experience, empathic concern) have been explored in a few studies. For example, JPOs with less than one year of work experience had significantly higher negative attitudes than those with more than 10 years of work experience (Ersayan et al., 2021). Decreased empathic concern (Copley et al., 2014) has been associated with negative attitudes. Negative attitudes toward offenders have also been found to influence POs’ supervisory strategies and, therefore, recidivism outcomes. Specifically, POs with more negative attitudes toward offenders employ stricter supervisory strategies for low‐risk offenders (Ricks et al., 2016). Such strict strategies have been documented as increasing recidivism risk (Oleson et al., 2011). In another study, punitive attitudes of JPOs were associated with increased recidivism risk perceptions (Ersayan et al., 2021). However, studies on the effects of these attitudes on these service professionals' burnout symptoms are rare, except for the studies by Gallavan and Newman (2013) and Schaefer and Williams (2018) mentioned before.

### 1.3 Demographic factors and job burnout: A mixed picture

Some research supports the relevance of years of work experience in explaining job stress and burnout symptoms. For instance, among 1741 Danish prison and probation service personnel, Andersen et al. (2017) found a negative linear association between years of work experience and job burnout. As seniority in the job increased, job burnout decreased. Contrary to this finding, Gayman et al. (2018) found positive correlations between years of job experience and emotional exhaustion among 893 parole/POs working for the North Carolina Department of Community Corrections. Interestingly, an earlier study by Whitehead (1985) corroborates this relationship pattern, because he found a curvilinear relationship between work experience and burnout in POs: the least and the most experienced POs reported lower burnout levels than those in the middle range of work experience.

Findings on the association between age and burnout are mixed as well. Age has shown both positive (Keinan & Malach‐Pines, 2007), and negative correlations (Maslach & Jackson, 1981) with job burnout among various human service professionals. Education level has also not been consistently correlated with burnout in correctional settings (Gayman & Bradley, 2013; Keinan & Malach‐Pines, 2007).

For a gender‐burnout link, prior research also provided little consistency in findings, although there appear to be differences in how men and women experience and/or express their job stress and burnout. In a study with 1025 human service workers (i.e., nurses, police officers, psychiatrists), Maslach and Jackson (1978) found that women experienced emotional exhaustion more frequently and more intensely than men. On the other hand, women scored lower than men on the frequency and intensity of the depersonalization and personal accomplishment dimensions of burnout. Purvanova and Muros (2010), in their meta‐analysis of gender differences in burnout, came to a similar conclusion, with women tending to experience higher levels of emotional exhaustion and men tending to experience higher levels of depersonalization.

### 1.4 The current study

The Turkish probation system was implemented in 2005 (Yavuz, 2012), and approximately 4000 POs manage more than 700,000 cases as of January 2020 (Turkish Ministry of Justice, 2020). A previous study documented medium to high levels of burnout among Turkish POs (Aslanyürek Zorlu, 2014). In a recent qualitative study, Turkish POs reported job burnout due to situational factors, such as too much paperwork, excessive caseloads, and role ambiguity (Erdem et al., 2019).

Thus far, the link between individual characteristics of POs and job burnout has not been studied in Turkey. Insight into these characteristics could be relevant for PO recruitment and may have implications for burnout prevention at the individual level. This insight could be especially relevant in a more recently established probation system, such as the one in Turkey because there is not yet a strong rehabilitative tradition in the probation service (Tuncer et al., 2020). The present study examines the association of individual factors with job burnout in Turkish POs. Specifically, we will investigate the link between attitudes toward probationers (ATP) and three dimensions of job burnout: Emotional exhaustion (EE), Depersonalization (DEP), and Professional accomplishment (PROF). In line with previous research among human service personnel, we hypothesize that more positive attitudes toward probationers will be related to less burnout reported by POs. Based on the available evidence (Gallavan & Newman, 2013; Schaefer & Williams, 2018), we presume that POs with less favorable attitudes toward their probationers will experience more struggle under their work conditions.

In light of the mixed findings concerning the association between demographic characteristics and job burnout (Andersen et al., 2017; Gayman & Bradley, 2013; Gayman et al., 2018; Keinan & Malach‐Pines, 2007; Maslach & Jackson, 1978, 1981; Purvanova & Muros, 2010; Whitehead, 1985) and the scarcity studies in non‐Western contexts, we will also explore the associations between gender, age, years of work experience, education level, and job burnout.

## 2 METHODS

### 2.1 Participants and procedure

We conducted the study in the Istanbul Probation Office, the largest probation office in Turkey. The criminal justice system in Turkey is centralized and the research site (as all other probation offices across the country) is managed by the Ministry of Justice. After obtaining official permission from the Turkish Ministry of Justice, we contacted the probation office and informed all staff about the study. Of 220 POs invited to the study, 116 agreed to participate (53% response rate). Those who did not participate in the study reported they were either on annual or maternity leave or had too much work.

Data were collected from June through October 2015. In order not to interfere with the busy work schedules of POs, we coordinated data collection with a site coordinator to schedule days for data collection and announce time, day, and room for the survey study ahead of time. Two research assistants visited the probation office once a week, informed officers about the study, and screened for eligibility. To be eligible for the study, the officers had to be working in the probation system for at least 6 months and have direct contact with probationers. After giving written consent, the officers filled out the questionnaires in a quiet room. The surveys took 20–25 min to complete. Because the participants were employed at a government institution, it was against the protocol to compensate them by monetary incentives. Instead, the participants had the option to attend either a social event with food and refreshments during lunch or a seminar about communication skills and stress management training as incentives. The present study was approved by the Turkish Ministry of Justice with protocol number 46985942‐773‐E.146/22516 and approved by Koç University's Institutional Review Board.

The sample included 30 (26%) women and 86 (74%) men. The mean age of officers was 28.14 years (*SD* = 4.89; range = 20–46 years). The average work experience in the probation system was 2.40 years (*SD* = 1.05; range = 1–5 years). The majority of the participants had at least a college degree (*n* = 70, 72%). The sample recruited through the Istanbul probation office in 2015 resembles the sex distribution of the Ministry of Justice staff nationwide. Turkish Ministry of Justice (2017) states that 27% of all probation and correctional staff are women. Of note, official statistics regarding the age and education level of POs have not been available since 2011.

### 2.2 Measures

Participants filled out a brief demographic form and reported their age, gender, educational level, and years of experience in the probation service.

*Maslach Burnout Inventory* (MBI; Maslach & Jackson, 1981). The MBI is a 22‐item 5‐point Likert‐type scale with three subscales. The *Emotional Exhaustion* subscale (EE; nine items) assesses the individual's level of depletion of emotional resources and inability to give to others. The *Depersonalization* subscale (DEP; five items) measures the aversion toward work, and the extent of negative feelings toward those one serves, in this case, probationers. The *Professional Accomplishment* subscale (PROF; eight items) taps into the feeling of effectiveness at work. Sample items are “I feel emotionally drained from work (EE), I don't really care what happens to my recipients” (DEP), and “I have accomplished many worthwhile things in this job” (PROF). The internal consistencies (Cronbach's *α*) of the EE, DEP, and PROF subscales of the MBI were 0.83, 0.65, and 0.72 when adapted and tested in Turkish healthcare workers (Ergin, 1996). In the current sample, subscale internal consistencies were 0.89, 0.74, and 0.78, respectively. A combination of high scores on EE and DEP and a low score on PROF corresponds to a high level of burnout.

*Attitudes Toward Prisoners Scale* (ATP; Melvin et al., 1985). The ATP is a 36‐item measure that assesses the strength of a person's negative attitudes toward prisoners. The items are rated on a 5‐point Likert‐type scale (from 1 = *disagree strongly* to 5 = *agree strongly*) with lower scores indicating more negative attitudes. Example items include “I wouldn't mind living next door to an ex‐prisoner.” and “Prisoners are no better or worse than other people.” The scale has been widely used to assess how different populations (i.e., social work students, correctional officers) perceive prisoners. It has shown high internal reliability (range = 0.84–0.92) and test–retest reliability (0.82; Melvin et al., 1985) when used with psychology students in the United States. The Turkish translation and psychometric studies of the ATP scale were conducted by Akdaş Mitrani et al. (2012) and showed high internal reliability (*α* = 0.92). For the current study, the scale was adapted to assess POs' attitudes toward their clients by replacing the word “prisoner” with “probationer.” The scale demonstrated high internal consistency in the current sample (*α* = 0.87).

### 2.3 Data analysis

We decided to perform Structured Equation Modeling to test out hypotheses and to probe the possible complexity of the interrelationships by exploring several path models that would allow us to examine the role of attitudes in relation to the various dimensions of job burnout while keeping demographic variables under control. For recursive models like ours, path analysis consists of a system of regression equations that can be solved with ordinary least squares estimation. Using Mplus version 7.2 for our path analyses, we obtained various fit indices that allowed us to identify the model that best described the observed covariance matrix. Model selection was based on an examination of the overall Chi‐square goodness‐of‐fit test, the value of the root mean square error of approximation (RMSEA; conventionally required to be smaller than 0.08 for a fair fit, and lower than 0.05 for a close fit), the Tucker Lewis Index (TLI; must be larger than 0.90 for an acceptable model) and the standardized root mean square residual for a direct indication of how well the original covariance matrix has been described by the model (SRMR; must be smaller than 0.08 for a close fit; Shi et al., 2019). Finding this optimal model also enabled us to attempt a replication of earlier results on the role of the demographic variables (PO's gender, age, years of work experience, and education level). In all path models, we examined the interrelationships between the three dimensions of job burnout as proposed by Taris et al. (2005) and based on an integration of earlier models proposed by Leiter and Maslach (1988), and Lee and Ashforth (1993). Guided by an analysis of longitudinal research with service providers, Taris et al.'s (2005) integrative model asserts a causal link with two main pathways: EE → DEP → PROF and EE → PROF. In addition to Taris et al.'s (2005) empirical research, we have conceptual reasons to expect such a causal model of three dimensions of burnout. Leiter and Maslach (1988) argue that EE is the main dimension, which triggers the burnout process, while DEP is a reactive and negative response to the exhaustion resulting from the overly demanding circumstances related to the job itself and the people within. PA, on the other hand, requires an overall evaluation of the work experience with direct and indirect links to the EE. We hypothesized that these pathways would be statistically significant in our models.

## 3 RESULTS

Table 1 gives an overview of mean scores and standard deviations for all variables in the model and their intercorrelations. For path models, outcome variables are assumed to be normally distributed (Bollen, 1989). Neither EE nor DEP showed significant skewness or kurtosis values. PROF was mildly left‐skewed (*skewness* = −0.48; *SE* = 0.23). Given the sample size, this was not considered problematic.

**Table 1:** Mean scores (with standard deviations in brackets) and Pearson correlations among predictor variables and three dimensions of job burnout

	Mean (*SD*)	1	2	3	4	5	6	7
1. Age	28.14 (4.89)							
2. Sex^a^	0.74 (0.44)	−0.08						
3. Work exp.^b^	2.29 (0.80)	***0.48*** c	**−0.20**					
4. ATP	3.38 (0.39)	−0.06	**−0.22**	0.16				
5. EE	1.81 (0.75)	−0.01	−0.01	0.06	−0.06			
6. DEP	1.35 (0.76)	−0.03	0.08	0.02	***−0.27***	***0.74***		
7. PROF.	1.32 (0.61)	−0.02	−0.17	0.04	**0.22**	−0.04	−0.08	

Abbreviations: ATP, attitudes toward probationers; DEP, depersonalization; EE, emotional exhaustion; PROF, professional accomplishment.

^a^ Sex is coded 0 for females and 1 for males. Mean of Sex reflects proportion of males in sample. Correlations with Sex are point‐biserial correlations.

^b^ Work experience proved very right‐skewed. We therefore merged the two most extreme categories “5 years” and “more than 5 years.” Means and correlations relate to the modified scale of work experience.

^c^ Correlation values printed in bold italics are significant at 0.01, values printed in bold but not in italics are significant at 0.05.

**Figure 1:**
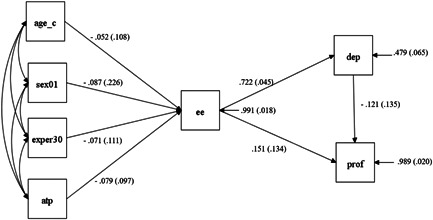
Model 1. All direct effects of continuous variables are standardized, with SE shown within parentheses. The effect of Sex01 is partially standardized. *Note:* Age has been expressed on a centered scale, Sex is a binary variable coded 0 for females and 1 for males, and Exper30 (referring to Work Experience) has been reverse‐coded with the most experienced group (≥5 years experience) as the reference category. ATP, attitudes toward probationers; DEP, depersonalization; EE, emotional exhaustion; PROF, professional accomplishment

Figure 1 shows the original path model that we used to describe the observed covariance matrix. Because EE is considered the key dimension of job burnout (Leiter & Maslach, 1988), all demographic variables, as well as ATP, were modeled to have an effect on EE, which [following Taris et al. (2005) model] was hypothesized to have an effect on both DEP and PROF. In addition, DEP was hypothesized to have a negative effect on PROF. Standardized path coefficients plus their corresponding standard errors are shown in Figure 1. Table 2 gives an overview of the fit indices for this model and for the two models that resulted from subsequent, sequential modifications.

**Table 2:** Fit indices for three path models

	Chi square (*df*)	*p*	TLI	RMSEA	SRMR
Model 1^a^	22.41 (8)	0.004	0.732	0.125	0.062
Model 2^b^	8.05 (7)	0.328	0.978	0.036	0.043
Model 3^c^	3.78 (7)	0.804	1.000	0.000	0.027

^a^ Model shown in Figure 1.

^b^ As Model 1, with addition of a direct effect of ATP on DEP.

^c^ As Model 2, with addition of a direct effect of ATP on PROF, and deletion of direct effect of ATP on EE, shown in Figure 2.

**Figure 2:**
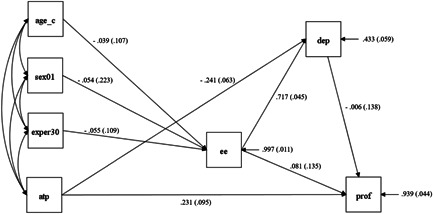
Final model

Dividing the standardized coefficients by their standard errors approximately yielded normally distributed *z*‐scores, with ratios larger than 2 (or smaller than −2) showing significant results. Model 1 provided a bad fit according to all goodness‐of‐fit indices [*χ* ^2^ (8) = 22.41, *p* = 0.004; *TLI* = 0.732; *RMSEA* = 0.125; *SRMR* = 0.062; see Figure 1]. Modification indices, which are chi‐square distributed with 1 df, showed the observed covariance between ATP and DEP to have been poorly reproduced. This led to the inclusion of ATP's direct effect on DEP in Model 2, resulting in an acceptable goodness‐of‐fit [*χ* ^2^ (7) = 8.05, *p* = 0.328; *TLI* = 0.978; *RMSEA* = 0.036; *SRMR* = 0.043; Figure not shown]. However, an inspection of the observed covariance matrix indicated further improvement by sequentially adding a direct effect of ATP on PROF and deleting the nonsignificant effect of ATP on EE. These additional modifications resulted in the final model, which indicated an almost perfect fit [*χ* ^2^ (7) = 3.78, *p* = 0.804; *TLI* = 1.0; *RMSEA* = 0.000; *SRMR* = 0.027; see Figure 2]. The final model shows that virtually none of the variance in EE can be explained by any exogenous variables (ATP and demographic variables).

Consistent with Taris et al.'s (2005) burnout model and prior empirical findings, most of the variance in DEP can be explained by its positive relationship with EE (*b* = 0.717; *SE* = 0.045) in our study. That is, EE tends to come with DEP. However, other hypothesized pathways are not statistically significant in our sample. Specifically, neither EE (*b* = 0.081; *SE* = 0.135) nor DEP (*b* = −0.006; *SE* = 0.138) shows a relationship with PROF.

Our results show that positive attitudes toward probationers tend to be associated with less DEP (*b* = −0.241; *SE* = 0.063) and show a clear positive relationship with PROF (*b* = 0.231; *SE* = 0.095). Contrary to our hypothesis, there is no association between ATP and EE (see Figure 2).

## 4 DISCUSSION

This study fills a gap in the current research field by examining several individual factors concerning job burnout levels experienced by POs within a non‐Western society. Among many challenges inherent in working within a correctional system, POs often carry a demanding caseload, receive poor support services, and often work toward unclear rehabilitative objectives (Holloway et al., 2013; Pitts, 2007; Steiner et al., 2004). Evidence to date suggests that such work conditions would increase the likelihood of job burnout (White et al., 2015; Whitehead, 1985). Similar to North American and European contexts, burnout, job stress, and role ambiguities are prevalent among Turkish POs (Altın, 2015; Erdem et al., 2019; Kamer, 2008), yet we know little about factors that contribute to burnout in POs. Such knowledge could help in designing interventions that would target system level, organizational changes, and perhaps more easily modifiable individual‐level changes, to support the mental health needs of POs.

Maslach et al. (2001) state that burnout is influenced by a multitude of factors that include not only work‐related conditions, but also individual‐level factors, such as attitudes. In this study, we investigated the association of attitudes toward probationers with the three separate components of job burnout. We hypothesized that disfavorable views about the people they serve (i.e., negative attitudes toward probationers) would significantly contribute to all dimensions of burnout, including emotional exhaustion, depersonalization, and professional accomplishment. Our hypotheses were confirmed for the associations of attitudes with depersonalization and personal accomplishment, but not emotional exhaustion.

Consistent with our expectations, more negative attitudes toward probationers were related to higher depersonalization. That is, officers seem to be more aversive to work and detached from probationers when they have such attitudes. The co‐occurrence of negative attitudes and depersonalization in our study may point at stigmatization of probationers. Goffman (1963) defines stigma as negative attitudes, emotional reactions, and behaviors toward a specific group based on its perceived characteristics. Distancing, detachment, and social exclusion are reported as indicators of stigma (McGinty et al., 2015; Nieweglowski et al., 2018). As such, negative attitudes toward and detachment from probationers may be indicators of stigma. Future studies could explore the experiences of probationers in the Turkish criminal justice system and examine potential indicators of stigmatization.

Another interpretation of the empirical link between depersonalization and negative attitudes could be found in the concept of coping with burnout. Depersonalization in itself may be viewed as a strategy to cope with the emotional burden of the work and thereby exacerbate negative attitudes toward probationers. Prior research has shown that Turkish POs, despite many complaints, feel hopeless about changing the probation system and report a lack of access to resources (Aslanyürek Zorlu, 2014; Erdem et al., 2019; Tuncer et al., 2020). That is, officers have little input into decision‐making and lack opportunities for training and supervision, factors that are significantly associated with depersonalization (Rhineberger‐Dunn & Mack, 2020). Within a context where POs do not see any alternatives to improving the services they provide, it is likely that they develop coping strategies that help them detach from probationers (in this case, depersonalization). Indeed, a similar study with POs (Wirkus et al., 2021) found that person‐aversive and avoidant coping strategies were preferred to cope with burnout and was perceived as adaptive, especially when opportunities such as career advancement were limited.

Additionally, we found that more positive attitudes toward probationers were linked to an increased sense of professional accomplishment among POs. This finding may indicate, though obviously speculative, that positive attitudes may function as an “intrinsic reward” for officers. In a similar vein, Finney et al. (2013) reported in a systematic review on burnout in correctional officers the important role of intrinsic rewards within the criminal justice system context, where extrinsic rewards are often limited.

Contrary to our hypothesis and prior research (e.g., Schaefer & Williams, 2018), we found attitudes toward probationers to be unrelated to emotional exhaustion. That is, negative attitudes were not associated with the depletion of emotional resources among POs. This finding could perhaps be attributable to the demographic makeup of our sample, which consisted mainly of men. There is evidence suggesting that men experience emotional exhaustion less frequently and less intensely compared to women (LaFaver et al., 2018; Maslach & Jackson, 1978). On the contrary, women experience depersonalization (Morgan et al., 2002; Savicki et al., 2003) and personal accomplishment less frequently and less intensely than men (Lambert et al., 2012). Thus, the lack of an association between negative attitudes and emotional exhaustion in the current sample might be due to our predominantly male sample (74%) as opposed to the study conducted by Schaefer and Williams (2018), where the majority of the sample was female (86%). These findings point to the relevance of gender in explaining job burnout symptoms. Besides, this finding also illustrates the utility of examining burnout in different countries due to potentially different staff demographic characteristics. The Turkish Ministry of Justice (2017) reports that 73% of staff are male in Turkey, contrary to its counterparts in Western countries.

Gender is not the only factor that could make a difference in what constitutes burnout across different contexts. According to the meta‐analysis of Purvanova and Muros (2010), the variation in the gender‐burnout link may be partially accounted for by differences in the work context, related to (conservative vs. progressive) labor policies. Accordingly, the female gender‐emotional exhaustion association was stronger for samples derived from countries with conservative labor policies^1^ (e.g., the United States) compared to samples derived from countries with progressive labor policies (e.g., Canada, EU). Turkey's labor policies belong to a neoliberal model closer to conservative labor policies (Sarımehmet Duman, 2014), and POs in the Turkish criminal justice system do not have unions to advocate for their labor rights (Yücel, 2017). Future research is needed to evaluate the link between labor policies and gender‐specific burnout symptoms in Turkey. Female POs constitute a minority of staff in the criminal justice system (Yücel, 2017); however, it would be pertinent to conduct a replication of our study in a predominantly female sample of Turkish POs, to investigate the replicability of our findings in female POs.

Another potential explanation for the lack of findings between exhaustion and burnout may relate to the overlap between reports of emotional exhaustion and depersonalization in the current sample (*r* = 0.74), showing that depersonalization may suppress a potential link between emotional exhaustion and attitudes. Gallavan and Newman's (2013) findings with correctional officers also hint at a conceptual link between emotional exhaustion and depersonalization. In their study, principal components analyses were conducted to operationalize burnout using the factors within the Maslach Burnout Inventory and Professional Quality of Life Survey (Stamm, 2009). In their analyses, emotional exhaustion and depersonalization along with secondary trauma stress loaded onto a component (named negative experience of work) whereas personal accomplishment and compassion satisfaction loaded onto another component (named positive experience of work). This finding indicates that emotional exhaustion and depersonalization share a more common variance compared to the variance between either of these dimensions and personal accomplishment. In Gallavan and Newman's (2013) study, attitudes toward prisoners were associated with positive experiences of work, but not with negative experiences of work. In sum, the operationalization of burnout, gender‐specificity, and context‐specific factors all play a role in the influence attitudes have on different dimensions of work‐related stress. Future research is warranted to inquire about the role of these influential factors in our understanding of burnout.

We also examined socio‐demographic correlates of burnout among POs. Hypotheses were not provided for the associations between socio‐demographic characteristics and levels of burnout because of conflicting findings in the literature (Andersen et al., 2017; Gayman & Bradley, 2013; Gayman et al., 2018; Keinan & Malach‐Pines, 2007; Maslach & Jackson, 1978, 1981; Purvanova & Muros, 2010; Whitehead, 1985). Our results pointed to a lack of associations between job burnout and demographic features.

In sum, the present study adds to the limited empirical knowledge base on how attitudes toward probationers relate to POs' psychological distress (Gallavan & Newman, 2013; Schaefer & Williams, 2018). Our results, except for the link between attitudes and exhaustion, are in line with those of Schaefer and Williams (2018), who found that positive attitudes were related to a reduced sense of depersonalization and an increased feeling of personal accomplishment.

### 4.1 Implications for future practice

Our findings may be of practical relevance in that interventions aimed at improving POs' attitudes toward probationers could help in enhancing the mental well‐being of POs. Correctional departments could take specific actions to increase resources for training and supervision for POs. First of all, these trainings could aim to increase mental health awareness and literacy among officers, to increase their understanding of the risk of burnout (White et al., 2015). According to our findings, POs’ attitudes toward probationers may be an individually modifiable target in reducing specific components of burnout. Having more positive attitudes toward probationers could help ensure a more positive work environment for POs (White et al., 2015), which according to our findings could not only help in increasing POs’ sense of professional accomplishment but also in decreasing their sense of detachment from probationers. The importance of attitudes toward probationers and their association with the risk of burnout could be a theme in professional educational training, individually tailored psychological interventions for POs and in supportive supervision to POs. Among psychological interventions, improvements in communication and interpersonal skills with probationers (Krasner et al., 2009), increasing coping skills, and cognitive‐behavioral treatments have been used in targeting burnout symptoms (Krasner et al., 2009; Maslach et al., 2001; Schaufeli & Peeters, 2000). In cognitive‐behavioral treatments, cognitive restructuring could be a viable target in decreasing dysfunctional and negative beliefs about probationers. In addition, an acceptance and values‐based workshop informed by Acceptance and Commitment Therapy has been shown to be effective in decreasing negative attitudes of counselors toward their substance‐abusing clients along with decreasing burnout rates (Hayes et al., 2004). White et al. (2015) suggested that this approach might be translated to workshops with POs. In light of the link between burnout and attitudes, we recommend that correctional departments consider organizational arrangements to allocate time and resources for educational and preventive workshops helping POs reformulate their perspective on their clients. This could not only improve the mental health burden of the work for POs but also improve the prospects of the probationers they serve.

Empirical evidence shows that attitudinal change in POs about their role in rehabilitative practices results in desirable outcomes for their probationers, such as reduced recidivism rates (Bourgon et al., 2011; Lowenkamp et al., 2013). The implementations of these interventions could be extended by attitudinal change theories (Wood, 2000), to target not only a shift in supervisory goals from repression to rehabilitation, but also perceptions of probationers. Previous research has shown positive associations between probationer success and improved interpersonal relationships between POs and probationers (e.g., collaborative communication; Kleinpeter et al., 2011; positive alliance; Clark et al., 2006). Future research could examine whether interventions that improve the quality of relationships between POs and probationers translate to more favorable attitudes toward probationers and decreased levels of job burnout (Gayman et al., 2018; Tuncer et al., 2020).

### 4.2 Limitations

The current study has several limitations. First, our study is cross‐sectional. Thus, causal interpretations cannot be made. Second, participants' responses were based on self‐report about their attitudes, personal and work characteristics, and experiences of burnout. Although confidentiality was ensured, we do not know whether participants answered honestly and/or whether fears of appearing incompetent impacted their responses. Third, our sample was composed of POs serving their duties in a metropolitan area in Turkey. For generalizable conclusions, future research needs to include a more extensive network of offices in the city or across the country. Fourth, most of the participants in our study were male. The majority of Turkey's POs are men (Yücel, 2017), and less is known about the work experiences of female staff in the probation system. Future research needs to cross‐validate our findings in a randomly selected sample of POs from different urban and rural regions of Turkey.

## 5 CONCLUSIONS

In comparison with relevant other research findings, our study did not provide evidence for the association of attitudes with all three dimensions of burnout. Our study demonstrates that there is a link between attitudes toward probationers and two dimensions of job burnout: depersonalization and professional accomplishment, but not emotional exhaustion. The current study contributes to the community psychology literature in several ways. Our study concerns the attitudes of officers employed in community‐based probation programs and their attributions about work experiences with probationers, a vulnerable and marginalized group in Turkish society. Additionally, the findings suggest areas of intervention, to diminish negative attitudes toward probationers and the risk of job burnout in these community service workers. Future research could examine contextual and community‐level factors that contribute to or intersect with officers’ mental health as well as attitudes toward probationers. Given the inconsistencies across findings from the few studies in this area and variations in measurement, there is also a need for future research that clarifies the psychological consequences of holding dis/favorable attitudes toward probationers in different countries.

### PEER REVIEW

The peer review history for this article is available at https://publons.com/publon/10.1002/jcop.22673

## CONFLICT OF INTERESTS

The authors declare that there are no conflict of interests.

## Footnotes

1 The United Nation International Labor Office (ILO) asks the member‐countries to adhere to the following policies: no more than 10 h of work per day including overtime, a weekly rest period of at least 24 h, at least 6 working days of annual paid vacation, which exclude public holidays, no less than 6 weeks of paid maternity leave per child, and compulsory health insurance for all employees. Whereas some countries such as the EU adopt policies that exceed the ILO standards, other countries such as the United States only meet the minimum recommended standards. The countries in the former group could be defined as countries with progressive labor policies. In contrast, countries in the latter group could be defined as countries with conservative labor policies (Purvanova & Muros, 2010).
